# CLE peptide-encoding gene families in *Medicago truncatula* and *Lotus japonicus*, compared with those of soybean, common bean and Arabidopsis

**DOI:** 10.1038/s41598-017-09296-w

**Published:** 2017-08-24

**Authors:** April H. Hastwell, Thomas C. de Bang, Peter M. Gresshoff, Brett J. Ferguson

**Affiliations:** 1 Centre for Integrative Legume Research, School of Agriculture and Food Sciences, The University of Queensland, St Lucia, Brisbane, Queensland 4072 Australia; 2 Plant Biology Division, Noble Research Institute LLC, Ardmore, Oklahoma 73401 USA; 3 Present Address: Department of Plant and Environmental Sciences, Section for Plant and Soil Sciences, Faculty of Science, University of Copenhagen, Thorvaldsensvej 40, DK-1871 Frederiksberg C, Denmark

## Abstract

CLE peptide hormones are critical regulators of many cell proliferation and differentiation mechanisms in plants. These 12-13 amino acid glycosylated peptides play vital roles in a diverse range of plant tissues, including the shoot, root and vasculature. CLE peptides are also involved in controlling legume nodulation. Here, the entire family of CLE peptide-encoding genes was identified in *Medicago truncatula* (52) and *Lotus japonicus* (53), including pseudogenes and non-functional sequences that were identified. An array of bioinformatic techniques were used to compare and contrast these complete CLE peptide-encoding gene families with those of fellow legumes, *Glycine max* and *Phaseolus vulgaris*, in addition to the model plant *Arabidopsis thaliana*. This approach provided insight into the evolution of CLE peptide families and enabled us to establish putative *M. truncatula* and *L. japonicus* orthologues. This includes orthologues of nodulation-suppressing CLE peptides and *AtCLE40* that controls the stem cell population of the root apical meristem. A transcriptional meta-analysis was also conducted to help elucidate the function of the CLE peptide family members. Collectively, our analyses considerably increased the number of annotated CLE peptides in the model legume species, *M. truncatula* and *L*. *japonicus*, and substantially enhanced the knowledgebase of this critical class of peptide hormones.

## Introduction

CLAVATA3/Endosperm Surrounding Region-related (CLE) peptides belong to a class of cysteine poor, post-translationally modified peptides that are derived from a prepropeptide^1–3^. The mature CLE peptide is 12 to 13 amino acids long and those that have been structurally confirmed all possess a tri-arabinose moiety attached to a highly conserved hydroxylated central proline residue^4–6^. They act as hormone-like signals^7^ and are perceived by class XI leucine-rich repeat receptor kinases^8^. They are also unique to plants, with the exception of CLE peptide-encoding genes of the cyst-knot nematode^9^, which were likely acquired from plants via horizontal gene transfer^6,10^. CLE peptides have roles in regulating stem cell populations of various plant organs^11,12^. Prominent examples include CLAVATA3 (CLV3) in the shoot apical meristem^13–15^, *AtCLE40* in the root apical meristem^16–18^, a number of legume-specific CLE peptides that suppress nodule organogenesis^2,19^, and a sub-class of highly conserved CLE peptides that regulate vascular differentiation^20–24^. Those of the cyst-knot nematode are thought to have a role in establishing the pathogen’s feeding site^25^.

*Medicago truncatula* and *Lotus japonicus* are model legume species that offer a number of molecular advantages to understanding aspects of legume development, as well as microbial and fungal symbioses^26^. However, only a few CLE peptide-encoding genes have been functionally characterised in these species to date. This includes *LjCLE-RS1*, *LjCLE-RS2*, *LjCLE-RS3*, *MtCLE12* and *MtCLE13*, which are involved in nodulation regulation^2,5,27–29^. Others include *LjCLE7*, *LjCLE15*, *LjCLE19 LjCLE20*, *LjCLE24* and *LjCLE29*, that are up-regulated in response to phosphate and/or mycorrhizae^30,31^; and *MtCLV3* ^32^ and *LjCLV3* ^27,33^, the orthologues of the most thoroughly characterised CLE peptide-encoding gene, *AtCLV3* ^15^. In *M. truncatula*, the likely orthologues of the Treachery Element Inhibitory Factor (TDIF) encoding genes, *AtCLE41*, *AtCLE42* and *AtCLE44* ^23,24^, have also been identified^3^.

Recent genomic and bioinformatic advances allow for the identification of entire peptide families. This is extremely helpful for comparable genomic studies and for advancing the important functional characterisation of individual peptide members. Here, we used a genome-wide approach to identify the complete CLE peptide-encoding gene families of *M. truncatula* and *L. japonicus*. Comparative bioinformatic approaches were used to assist in identifying orthologous genes between these, and other plant species, as well as in the categorisation and functional characterisation of these critical peptide-encoding genes.

## Results

### Identification of CLE peptide-encoding genes in *L*. *japonicus* and *M*. *truncatula*

**Figure 1:**
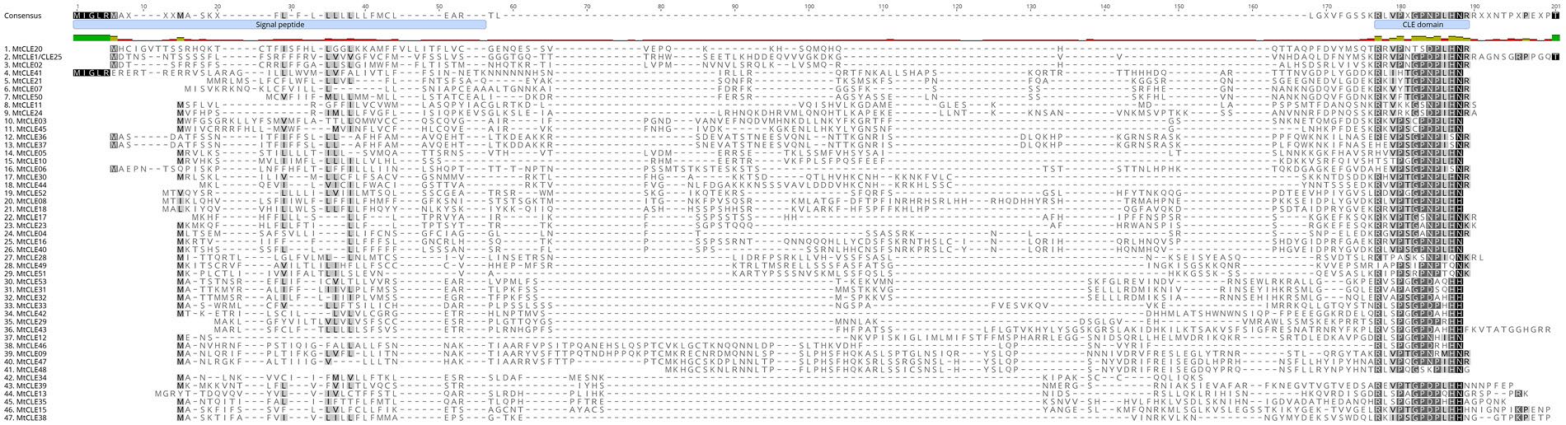
Multiple sequence alignment of *Medicago truncatula* CLE prepropeptides. The sequences show high similarity, as indicated by darker shading, in the signal peptide and CLE domains. Not shown are the multi-CLE domain containing prepropeptides (MtCLE14, MtCLE26, MtCLE27 and MtCLE22, see Fig. 3) and MtCLE19, which has a premature stop codon very early in the prepropeptide (see Fig. 4). MtCLE34 is a likely pseudogene without a functional CLE domain. The signal peptide approximate location and CLE domain is shown on the consensus sequence.

**Figure 2:**
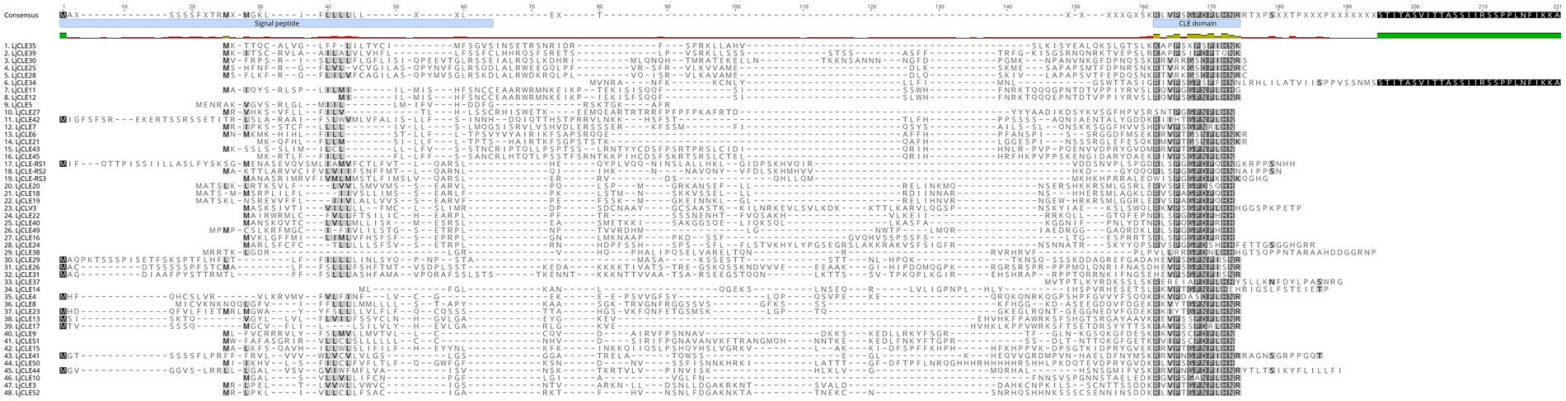
Multiple sequence alignment of *Lotus japonicus* CLE prepropeptides. As with the *M. truncatula* sequences (Fig. 1), the *L. japonicus* sequences show high similarity in the signal peptide and CLE domains, as indicated by darker shading. Not shown are the multi-CLE domain containing prepropeptides (LjCLE32, LjCLE33, LjCLE46 and LjCLE47; see Fig. 3) and LjCLE48, the truncated *L. japonicus* AtCLE40 orthologue as it shows very little amino acid conservation. LjCLE5 is a likely pseudogene without a functional CLE domain (see Fig. 5). The signal peptide approximate location and CLE domain is shown on the consensus sequence.

**Figure 3:**
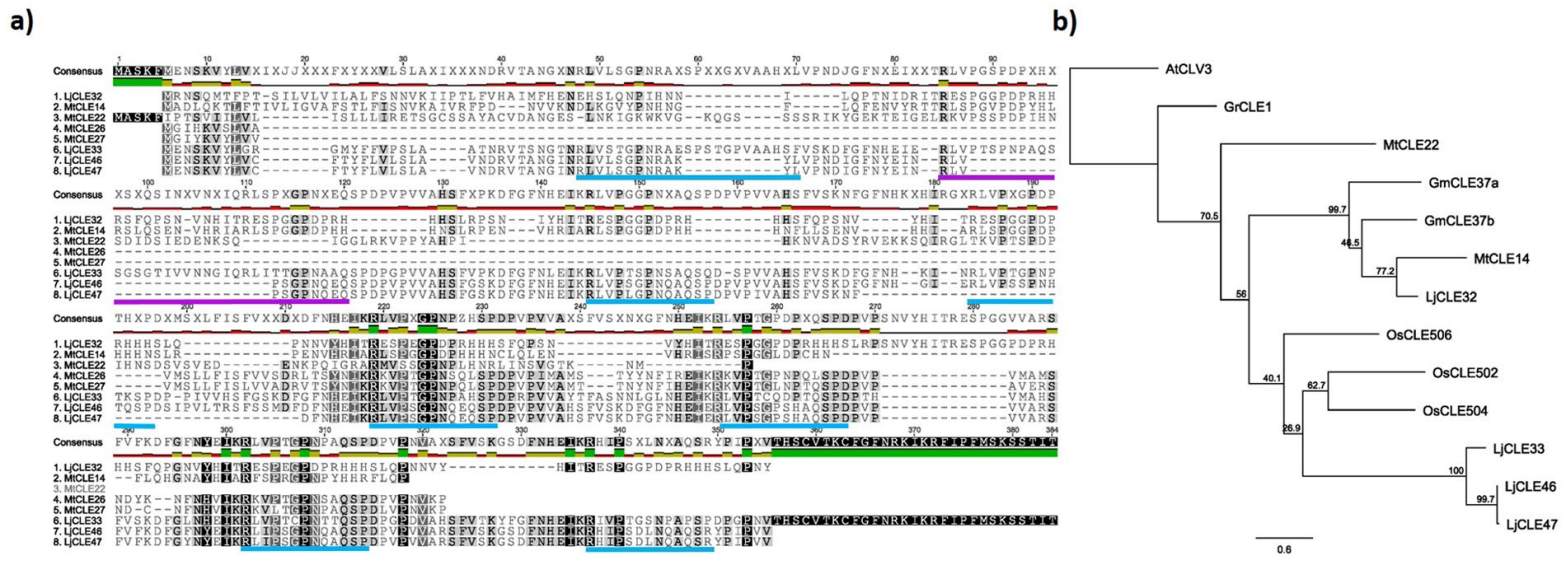
Multi-CLE domain prepropeptides. (**a**) Multiple sequence alignment of four *Lotus japonicus* (LjCLE32, LjCLE33, LjCLE46 and LjCLE47) and four *Medicago truncatula* (MtCLE14, MtCLE26, MtCLE27 and MtCLE22) multi-CLE domain containing prepropeptides (See Supplementary Table S3). Putative CLE domains are located above the blue and purple underlined regions. LjCLE21, LJCLE33 and MtCLE14 also have a second CLE domain present above the purple underlined region. (**b**) Phylogenetic tree of known multi-CLE domain prepropeptides in *L. japonicus*, *M. truncatula*, *Glycine max*, *Oryza sativa* and potato cysts nematode (*Globodera rostochiensis*), including AtCLV3 as an outgroup. The tree is shown with bootstrap confidence values as a percentage of 1,000 bootstraps.

A thorough genome-wide search of the *M. truncatula* and *L. japonicus* genomes was conducted to identify the complete CLE peptide-encoding gene families of these species. Multiple BLAST searches identified 52 and 53 CLE peptide-encoding genes in each of the two species respectively (Figs 1–3, Table 1). Initial BLAST and TBLASTN queries used sequences of known soybean and *A. thaliana* CLE peptide-encoding genes and prepropeptides^3^ to ensure all genes of interest were captured. The resulting identified sequences were verified and false-positives removed from further analyses. Additional CLE peptide-encoding genes were identified by BLAST and TBLASTN reciprocal searches of the *M. truncatula* and *L. japonicus* genomes using the sequences identified in the initial searches. A number of the genes identified are reported here for the first time, with the nomenclature of the newly discovered genes consistent with previously identified CLE peptide-encoding genes (Figs 1–3, Table 1). A recent study published after our searches were conducted included 20 *M. truncatula* CLE peptide-encoding genes (Goad *et al*., 2016), but no nomenclature was given as species-specific analyses were not conducted. A complete listing of all CLE peptide encoding gene family members from *M. truncatula* and *L. japonicus* is provided in Supplementary Table S1.

**Table 1:** Name, ID and various features of CLE genes in *Medicago truncatula* and *Lotus japonicus*.

Name	Phytozome V11 ID (Mtv4)	Pre-propeptide length	Chromosome location	Orientation	Predicted intron	SP cleavage site^a^
MtCLE1/CLE25	Medtr5g037140	110	chr5:16209190..16209492	forward	No	39
MtCLE02	Medtr6g009390	90	chr6:2758371..2758643	reverse	No	34
MtCLE03	Medtr1g110820	99	chr1:50033208..50033507	forward	No	31
MtCLE04	Medtr5g014860	66	chr5:5053422..5053622	reverse	No	26
MtCLE05	Medtr1g100733	84	chr1:45667039..45667293	forward	No	25
MtCLE06	Medtr7g058790	99	chr7:21150139..21150438	reverse	No	35
MtCLE07	Medtr7g089320	85	chr7:34939800..34940057	forward	No	28
MtCLE08	Medtr8g076990	120	chr8:32679901..32680263	reverse	No	27
MtCLE09	Medtr7g084110	137	chr7:32430490..32430903	reverse	No	31
MtCLE10	Medtr6g054925	74	chr6:19620161..19620385	forward	No	27
MtCLE11	Medtr3g037730	83	chr3:13874060..13874311	reverse	No	27
MtCLE12	Medtr4g079630	81	chr4:30800344..30800589	forward	No	29
MtCLE13	Medtr4g079610	84	chr4:30793797..30794051	forward	No	27
MtCLE14	Medtr7g084100	221	chr7:32428499..32429164	reverse	No	28
MtCLE15	Medtr2g087170^b^	100	chr2:36639989..36640517	forward	Yes	26
MtCLE16	Medtr5g043830	101	chr5:19252630..19252935	reverse	No	20
MtCLE17	Medtr5g085990	72	chr5:37176921..37177139	reverse	No	21
MtCLE18	Medtr1g093800	104	chr1:42088638..42088952	forward	No	27
MtCLE19	unannotated	c	chr7:46832505..46832810	forward	No	ND
MtCLE20	Medtr1g018700	91	chr1:5449791..5450066	forward	No	43
MtCLE21	Medtr5g089080	84	chr5:38716369..38716623	forward	No	27
MtCLE22	Medtr2g087180	181	chr2:36645611..36646743	reverse	Yes	26
MtCLE23	Medtr3g080060	72	chr3:36207269..36207487	forward	No	24
MtCLE24	Medtr5g040640	108	chr5:17857512..17857838	reverse	No	27
MtCLE26	unannotated	117	chr1:27528684..27529037	forward	No	22
MtCLE27	Medtr1g062850	116	chr1:27531440..27531790	forward	No	22
MtCLE28	Medtr1g106920	89	chr1:48060869..48061138	forward	No	23
MtCLE29	Medtr2g015445	78	chr2:4575877..4576113	forward	No	24
MtCLE30	Medtr2g038665	81	chr2:16905712..15905957	reverse	No	22
MtCLE31	Medtr2g078130	85	chr2:32437536..32437793	reverse	No	26
MtCLE32	Medtr2g078140	83	chr2:32444505..32444756	reverse	No	22
MtCLE33	Medtr2g078160	75	chr2:32460085..32460212	reverse	No	22
MtCLE34	Medtr2g091120	49	chr2:39236945..39237094	forward	No	23
MtCLE35	Medtr2g091125	92	chr2:39246810..39247088	forward	No	24
MtCLE36	Medtr2g437780	108	chr2:14915100..14915427	forward	No	27
MtCLE37	Medtr2g437800	108	chr2:14923591..14923917	forward	No	27
MtCLE38	Medtr4g051618	75	chr4:18583841..18584193	reverse	Yes	22
MtCLE39	Medtr4g066100	82	chr4:24906117..24906560	reverse	Yes	21
MtCLE40	Medtr4g082920	92	chr4:32235471..32235749	reverse	No	25
MtCLE41	Medtr4g084520	114	chr4:32892006..32892350	reverse	No	32
MtCLE42	Medtr4g087850	74	chr4:34563502..34563726	reverse	No	23
MtCLE43	unannotated	117	chr4:34572155..34572508	reverse	No	22
MtCLE44	Medtr4g126940	89	chr4:52604820..52605086	reverse	No	19
MtCLE45	Medtr5g056935	84	chr5:23427594..23427848	reverse	No	32
MtCLE46	Medtr7g084130	98	chr7:32437149..32437445	reverse	No	27
MtCLE47	unannotated	126	chr7:32590148..32590528	reverse	No	28
MtCLE48	unannotated	87	chr7:32594246..32594509	reverse	No	ND
MtCLE49	Medtr7g093050	92	chr7:36960868..36961146	reverse	No	27
MtCLE50	Medtr7g094080	81	chr7:37442023..37442268	forward	No	24
MtCLE51	Medtr8g042980	78	chr8:16651803..16652039	forward	No	20
MtCLE52	Medtr8g096970	89	chr8:40711987..40712256	reverse	No	25
MtCLE53	Medtr8g463700	82	chr8:22459174..22469422	forward	No	26
LjCLE3	Lj4g3v2140240.1	81	chr4:29668322..29668567	forward	No	19
LjCLE4	Lj5g3v0296280.1	87	chr5:2776799..2777062	reverse	No	28
LjCLE5	Unannotated	37	chr0:191639..191752	reverse	No	14
LjCLE6	Lj2g3v2904560.1	77	chr2:37869993..37870226	reverse	No	28
LjCLE7	Lj5g3v2013980.1	85	chr5:28436402..28436659	forward	No	26
LjCLE8	Lj1g3v4106880.1	90	chr1:48730186..48730458	forward	No	29
LjCLE9	Lj2g3v1155200.1	84	chr2:18195792..18196046	reverse	No	30
LjCLE10	Lj2g3v1984000.1	58	chr2:28901709..28901885	reverse	No	20
LjCLE11	Lj4g3v2917660.1	91	chr4:38847122..38847397	reverse	No	28
LjCLE12	unannotated	78	chr4:38846082..38846318	reverse	No	ND
LjCLE13	Lj5g3v1494620.1	77	chr5:21653524..21653754	reverse	No	31
LjCLE14	Lj3g3v1261020.1	71	chr3:16238530..16238742	forward	No	ND
LjCLE15	Lj5g3v1789230.1	104	chr5:25374552..25374863	forward	No	27
LjCLE16	Lj6g3v1996000.1	75	chr6:23228716..23228940	reverse	No	21
LjCLE17	Lj1g3v4931750.1	74	chr1:60060788..60061012	forward	No	27
LjCLE18	Lj3g3v1063710.1	80	chr3:14363293..14363535	reverse	No	26
LjCLE19	Lj3g3v0428680.1	84	chr3:4038516..4038770	reverse	No	28
LjCLE20	Lj3g3v0428740.1	83	chr3:4052954..4053205	reverse	No	27
LjCLE21	Lj6g3v1055570.1	73	chr6:12069820..12070041	reverse	No	22
LjCLE22	Lj0g3v0114139.1	76	chr0:49962922..49963152	forward	No	23
LjCLE23	Lj0g3v0005899.1	96	chr0:2220612..2220902	reverse	No	35
LjCLE24	Lj4g3v0496580.1	110	chr4:8347397..8347729	forward	No	22
LjCLE25	Lj4g3v1635250.1	94	chr4:24032504..24032788	reverse	No	27
LjCLE26	Lj4g3v0189810.1	122	chr4:2377271..2377630	forward	No	33
LjCLE27	Lj2g3v0276540.1	86	chr2:4685035..4685295	forward	No	21
LjCLE28	Lj1g3v0492090.1	95	chr1:6477403..6477690	forward	No	20
LjCLE29	Lj2g3v1389560.1	99	chr2:22031058..22031357	forward	No	40
LjCLE30	Lj2g3v1277900.1	109	chr2:20511088..20511417	forward	No	23
LjCLE31	Lj6g3v1415960.1	124	chr6:16797110..16797364	reverse	No	31
LjCLE32	Unannotated	274	chr1:45492391..45493215	reverse	No	27
LjCLE33	Lj3g3v1314940.1	347	chr3:17115763..17116806	forward	No	ND
LjCLE34	Lj3g3v2248290^b^	89	chr3:126894445..126894714	forward	No	26
LjCLE35	Unannotated	79	chr5:30013235..30013474	forward	No	19
LjCLE37	Unannotated	44	chr6:16475826..16475960	reverse	No	ND
LjCLE38	Lj1g3v4241120.1	77	chr1:50124894..50125127	reverse	No	ND
LjCLE39	Lj1g3v4317570.1	93	chr1:50801119..50801398	reverse	No	29
LjCLE40	Lj3g3v2848710.1	80	chr3:35016355..35016597	forward	No	22
LjCLE41	Lj2g3v1354640.1	98	chr2:21357987..21358244	forward	No	32
LjCLE42	Lj4g3v0643890.1	111	chr4:10320058..10320393	forward	No	41
LjCLE43	Unannotated	96	chr0:78808582..78808872	forward	No	24
LjCLE44	Lj2g3v1022600.3	100	chr2:16174090..16174392	reverse	No	29
LjCLE45	Lj2g3v1265080.1	96	chr2:20113738..20114028	forward	No	21
LjCLE46	Lj3g3v1314910.1	285	chr3:17104803..17105660	reverse	No	27
LjCLE47	Lj3g3v1314920.1	250	chr3:17108302..17109054	reverse	No	27
LjCLE48	Unannotated	c	chr3:40213173..40213683	forward	Yes^c^	ND
LjCLE49	Lj4g3v0496650.1	74	chr4:08364934..08365729	forward	No	26
LjCLE50	Lj4g3v1785920.1	102	chr4:25243942..25244250	reverse	No	25
LjCLE51	Lj5g3v2193950.1	98	chr5:31824885..23948823	forward	No	30
LjCLE52	Lj6g3v1280830.1	82	chr6:15475896..15476144	reverse	No	19
LjCLE-RS1	Lj0g3v0000559.1	116	chr0:240796..241074	reverse	No	23
LjCLE-RS2	Lj3g3v2848800.1	81	chr3:35039780..35040025	forward	No	28
LjCLE-RS3	Lj3g3v2848810.1	72	chr3:35048895..35049113	forward	No	28
LjCLV3	Lj3g3v1239970.1	105	chr3:16182605..16182777	forward	Yes	24

^a^Signal peptide cleaved after noted residue. ^b^Unannotated transcript variant. ^c^Likely untranscribed pseudogene. ND, not detected.

Additional CLE peptide-encoding genes in both *L. japonicus* and *M. truncatula* were identified that contain multiple CLE domains; some of which are also reported here for the first time. These multi-CLE peptide domain encoding genes include *LjCLE32*, *LjCLE33, LjCLE46* and *LjCLE47* in *L. japonicus*; and *MtCLE14*, *MtCLE22*, *MtCLE26* and *MtCLE27* in *M. truncatula* (Fig. 3). *LjCLE32* and *LjCLE33* encode eight and nine putative CLE peptides respectively; *MtCLE22* encodes four putative CLE peptides; *MtCLE26* and *MtCLE27* encode three putative CLE peptides; whereas all others contain seven putative CLE peptide domains (Fig. 3a; Supplementary Table S1). Interestingly, these multi-CLE domain containing genes contain repeating motifs of 24 to 35 amino acids, with each motif having a consistent length within their respective prepropeptide, with the sole exception of LjCLE33 which has varying motif lengths (Supplementary Table S2).

**Figure 4:**
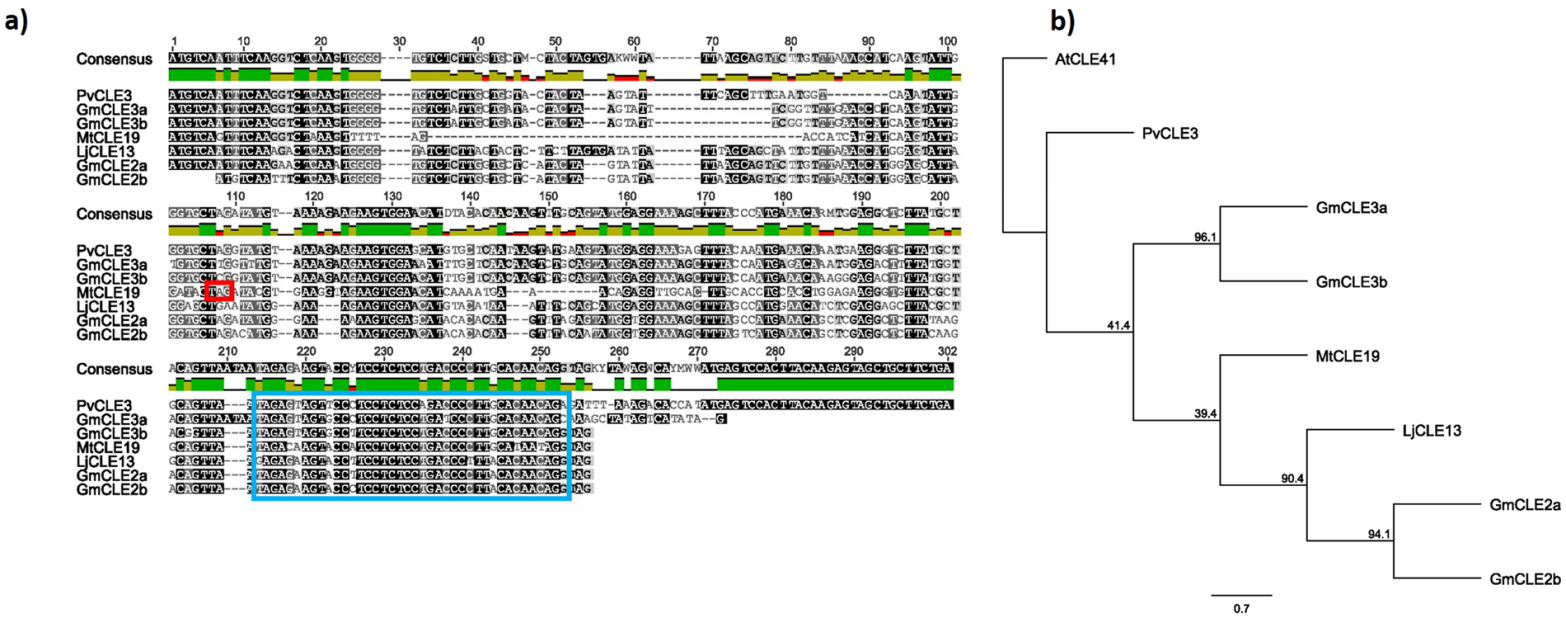
Genomic sequence characterisation of *MtCLE19*, the likely non-functional *M. truncatula* orthologue of *GmCLE2a*, *GmCLE2b*, and *LjCLE13*. (**a**) Multiple sequence alignment demonstrating that *MtCLE19* exhibits high similarity to *GmCLE2a*, *GmCLE2b*, and *LjCLE13*, with slightly less similarity to *GmCLE3a*, *GmCLE3b* and *PvCLE3*. The red box indicates a premature stop codon and the blue box indicates the CLE domain. Grey nucleotides are semi-conserved and black nucleotides are 100% conserved. (**b**) Phylogenetic tree with bootstrap confidence values expressed as a percentage of 1,000 bootstrap replications, using *AtCLE41* as an outgroup.

**Figure 5:**
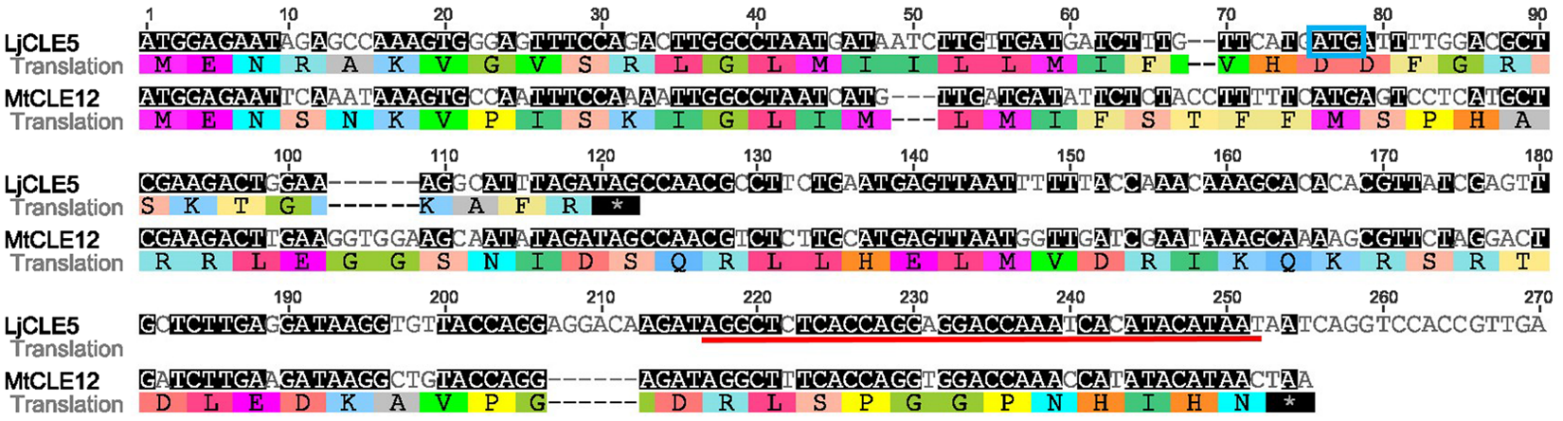
Multiple sequence alignment of the prepropeptides of AtCLE18 and LjCLE34. CLE domains are highlighted with a red box and the CLEL domain is underlined in blue. Conservation between amino acid residues of the two sequences is represented by grey (partial) and black (100%) shading.

**Figure 6:**
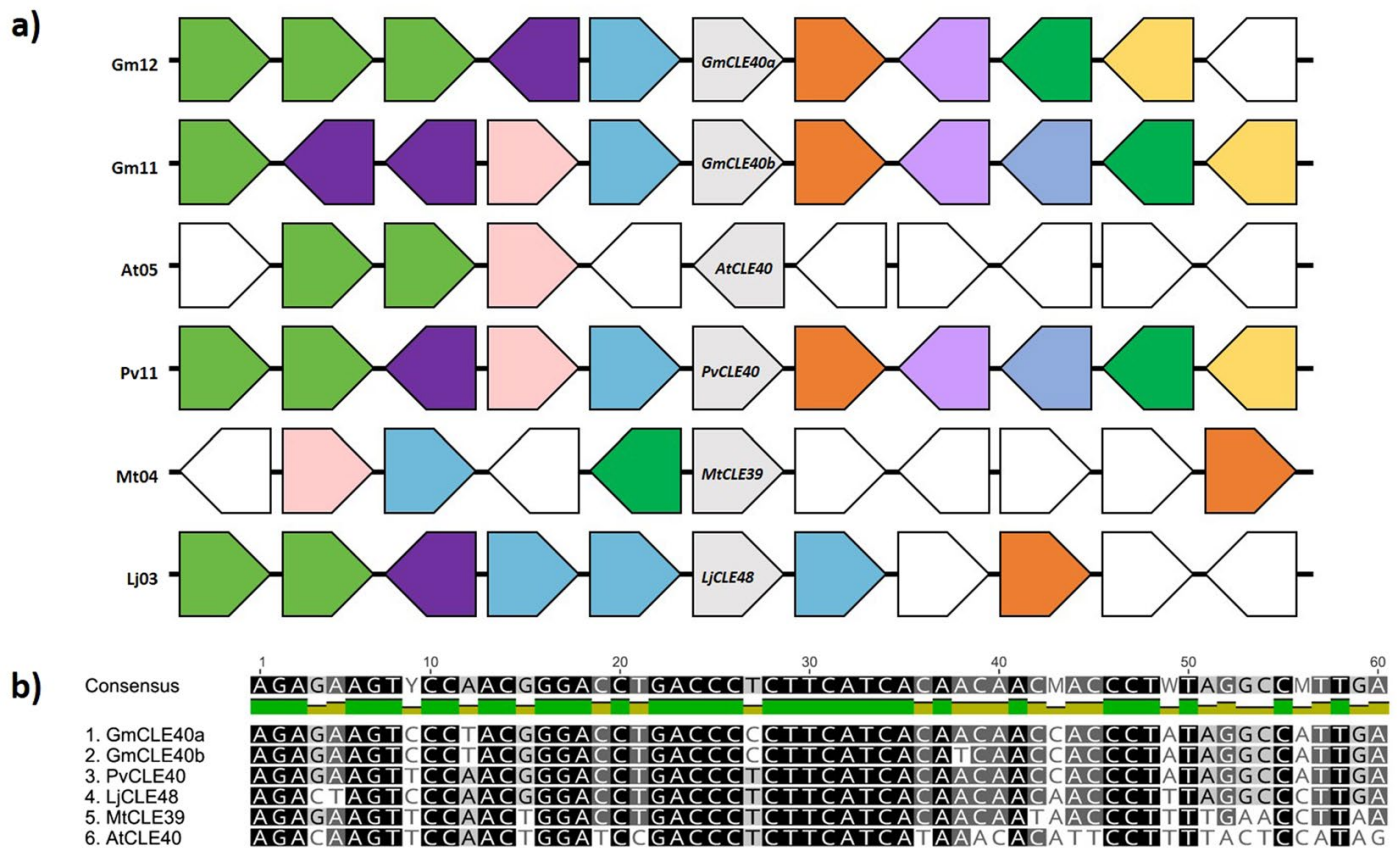
*AtCLE40* and orthologues in *Medicago truncatula*, *Phaseolus vulgaris*, and *Glycine max*, in addition to the truncated orthologue *in Lotus japonicus*, LjCLE48. (**a**) The genomic environment of each shows strong synteny. Arrows represent individual genes and their transcriptional direction in relation to *CLE40*. Similar colours represent genes from the same family, and are typically orthologous. (**b**) A multiple sequence alignment of the CLE40 domain coding region. Shading represents conservation amongst nucleotides with grey nucleotides semi-conserved and black nucleotides 100% conserved.

Pseudogenes were also identified in both the *L. japonicus* and *M. truncatula* genomes. These genes include mutations where the CLE domain is not translated in frame, likely resulting in a non-functional gene. This includes the pseudogenes *MtCLE34*, which is annotated within the *M. truncatula* genome (Fig. 1, Table 1; Supplementary Fig. S1) and *MtCLE19* (Fig. 4). In *L. japonicus*, LjCLE5 (Figs 2 and 5, Table 1) and LjCLE48 are also unlikely to be functional (Fig. 6). These pseudogenes, and the genes containing multiple CLE-domains, were excluded from the sequence characterisation studies detailed below because they fail to align well with the more typical single-CLE domain sequences.

A BLAST search of the *L. japonicus* genome with the *LjCLE34* nucleotide sequence (first reported by Okamoto *et al*.^27^), identified two possible genes having two synonymous nucleotide changes that result in identical prepropeptides. These genes are located at chr3:27855838..27856107 and chr0:126894445..126894714, and interestingly, both are found within a larger predicted protein. It therefore appears that these two genes arose as a transposable element and subsequent duplication event, or they are the result of a genome sequencing error. Interestingly, the CLE domain of LjCLE34 is not located at the C-terminus of the prepropeptide but towards the centre, similar to that of AtCLE18, which has a C-terminal CLE-Like/Root Growth Factor/GOLVEN (CLEL/RGF/GLV) domain in addition to a CLE domain^34^. LjCLE34 shares some homology at the C-terminus with AtCLE18 which includes the region of the CLEL/RGF/GLV domain (Supplementary Fig. S2).

CLE peptide-encoding genes of *M. truncatula* and *L. japonicus* are located across all chromosomes, with the greatest number located on chromosome two of *M. truncatula* (eleven) and chromosome three of *L. japonicus* (thirteen) (Table 1). There are five CLE peptide-encoding genes of *L. japonicus* currently located on unassigned scaffolds (Table 1). The CLE prepropeptides of both species vary in length, with the average single-CLE domain prepropeptide being 88 residues in *L. japonicus* and 91 residues in *M. truncatula*. The multi-CLE domain prepropeptides of both species range from 116 to 347 amino acids.

Some CLE peptide-encoding genes appear directly in tandem within the genome. For example, on chromosome 2 of *M. truncatula*, *MtCLE31* is 6.7 Kb upstream of *MtCLE32*, which itself is 15.3 Kb upstream of *MtCLE33*. Also on chromosome 2, *MtCLE34* is 9.6 Kb upstream of *MtCLE35* and *MtCLE36* is 6.7 Kb upstream of *MtCLE37*. On chromosome 7, *MtCLE14*, *MtCLE09* and *MtCLE46* are all within 9 Kb, and *MtCLE47* is 3.7 Kb upstream of *MtCLE48*. On chromosome 4, *MtCLE12* and *MtCLE13* are not directly in tandem, but are only 6.3 Kb apart (Table 1). On chromosome 3 of *L. japonicus*, *LjCLE46* is 2.6 Kb apart from *LjCLE47*, which is 6.7 Kb upstream of *LjCLE33*. Also on chromosome 3, *LjCLE40*, *LjCLE-RS2* and *LjCLE-RS3* are within 24 Kb, and although not directly in tandem, *LjCLE19* and *LjCLE20* are only 14.2 Kb apart. On chromosome 4, *LjCLE11* and *LjCLE12* are only 0.8 Kb apart (Table 1). Interestingly, the genes appearing directly in tandem within the *L. japonicus* genome share >50% amino acid sequence similarity, while only some of the tandem gene pairs in *M. truncatula* exhibit more than a 50% level of similarity (Supplementary Table S3).

### Identification of orthologous CLE peptide sequences

To identify gene orthologues of the *M. truncatula* and *L. japonicus* CLE prepropeptides, multiple sequence alignments were generated. Most orthologues were present in a 1:1 ratio between the two species (Supplementary Fig. S3). When no orthologue was evident, further BLAST searches were conducted in an attempt to identify one. In some instances, this yielded additional CLE peptide-encoding genes. Subsequent multiple sequence alignments with the CLE prepropeptides of *M. truncatula*, *L. japonicus*, soybean, common bean and *A. thaliana* were constructed (data not shown) and used to identify additional CLE peptide-encoding genes. All orthologous sequences identified are shown in Figs 1 and 2.

A multiple sequence alignment of the prepropeptides of *M. truncatula*, *L. japonicus*, common bean and *A. thaliana* was used to construct a phylogenetic tree (Supplementary Fig. S3). Similar phylogenetic trees have been constructed using only the CLE domain of the prepropeptides; however, this domain is highly conserved and only 12-14 amino acids long, and hence alignments and trees constructed using only the conserved motif can be less informative. In contrast, the tree constructed here, using the entire prepropeptide sequences, allows for the identification of conserved residues within other domains that may relate to cleavage and other important facets of post-translational modification^2^.

### Characterisation of *M*. *truncatula* and *L*. *japonicus* CLE prepropeptides

The domain structure of all CLE prepropeptides includes a hydrophobic signal peptide near the N-terminus, followed by a large variable region and a short but highly conserved CLE domain (with a multi-CLE domain occasionally present) and a small number (11 in *L. japonicus* and 8 in *M. truncatula*) that have a short C-terminal extension of unknown function (Figs 1 and 2)^2^. The amino acid composition of all known CLE prepropeptides, across legume and non-legume species, is typically rich in lysine and serine, and poor in tyrosine, cysteine and tryptophan, with the latter being poorly represented in all plant proteins^3^. The CLE prepropeptides of *M. truncatula* and *L. japonicus* fit this amino acid profile (Supplementary Table S4). The CLE domain represents the functional peptide ligand, which is post-translationally cleaved and modified to 13 amino acids in AtCLV3 and LjCLE-RS1^4–6,35^. A total of 66% (*L. japonicus*) and 61% (*M. truncatula*) of the prepropeptides have an amino acid at the 13^th^ residue, with the remaining having a stop codon at position 13, and thus being only 12 amino acids long. In both species, the amino acid most commonly found at position 13 is arginine (Figs 1 and 2, Supplementary Fig. S4).

An arginine residue is found at the start of 83% of *L. japonicus* and 87% of *M. truncatula* CLE domains. Although less common, a number of CLE domains also begin with a histidine, and this is conserved between orthologues of different species. Three of the four peptides beginning with a histidine in *A. thaliana* are Tracheary Differentiation Inhibitory Factors (TDIF) that are involved in vascular differentiation^36^. *L. japonicus* and *M. truncatula* each have three CLE peptides beginning with a histidine (LjCLE26, LjCLE29 and LjCLE31, and MtCLE05, MtCLE06 and MtCLE37) that appear orthologous to the TDIF factors. However, they do not appear to have an orthologue of the functionally unrelated fourth CLE peptide of Arabidopsis to begin with a histidine, AtCLE46, and its putative soybean orthologue, GmCLE13^3^.

The most highly conserved CLE domain residues of *M. truncatula* are arginine at position one, glycine at position six and histidine at position 11, with all three present in 87% of the peptides (Fig. 1). Interestingly, the most conserved CLE domain residue of *L. japonicus* is histidine at position 11 (91%), with only three sequences having a serine at this position and one sequence having a glutamine (Fig. 2). Residues 1, 4, 6, 7, 9 and 11 are also highly conserved (>82%) in the CLE domain of both species (Figs 1 and 2, Supplementary Fig. S4). These residues are all considered critical for function except for the proline at position nine^37^.

Outside of the CLE domain there is little conservation within the *L. japonicus* and *M. truncatula* CLE prepropeptide families (Figs 1 and 2). However, the signal peptide, which is predicted to either export the entire prepropeptide or the cleaved propeptide outside of the cell^1,38^, contains a typical hydrophobic motif consisting of predominantly leucine and isoleucine (Figs 1 and 2). The size of the predicted signal peptide ranges from 19 to 43 residues (Table 1). Additionally, the truncated LjCLE5 prepropeptide has a predicted signal peptide cleavage site between residues 14 and 15 (Table 1).

Hastwell *et al*.^3^ classified the CLE prepropeptides of soybean and common bean into seven distinct Groups (I to VII). The prepropeptides within each group show sequence conservation within and outside of the CLE domain. Based on the phylogenetic tree of the prepropeptides in *L. japonicus*, *M. truncatula*, *A. thaliana* and *P. vulgaris*, these groups remain conserved (Supplementary Fig. S3, Supplementary Table S5). This is especially evident with the Group VI CLE prepropeptides, which function in nodulation regulation, and Group III CLE prepropeptides, which show high sequence conservation with the Arabidopsis TDIF peptides, AtCLE41, AtCLE42 and AtCLE44 (Supplementary Fig. S3, Supplementary Table S5).

### Identification of CLE40

A well characterised peptide, AtCLE40, has been shown to act as the root paralogue of AtCLV3 to regulate the stem cell population of the root apical meristem^16–18^. Putative orthologues of *AtCLE40* have been identified in *M. truncatula*, *P. vulgaris* and *G. max* (*MtCLE39*, *PvCLE40*, *GmCLE40a* and *GmCLE40b* ^3^). Interestingly, our BLAST searches using the *L. japonicus* genome failed to identify a CLE40 orthologue. However, a region on chromosome 3 (chr3:40213173..40213683) exhibits a very high level of sequence similarity to these *CLE40* orthologues, in addition to having a similar genomic environment to them (Fig. 6). All previously identified *CLV3* and *CLE40* orthologues contain two introns. The putative *L. japonicus CLE40* orthologue, identified here as *LjCLE48*, contains conserved predicted intron boundaries for the second intron, which correspond to the *CLE40* orthologues, but there are no predicted boundary sites for the first intron. Given this critical change at the 5′ end of *LjCLE48*, it appears unlikely that the resulting prepropeptide would produce a functional peptide product. This may suggest that another CLE peptide has evolved to perform the function of CLE40 in *L. japonicus*.

### Nodulation CLE peptides

CLE genes in Group VI of soybean and common bean are known to respond to symbiotic bacteria, collectively called rhizobia, and act to control legume nodulation. The rhizobia-induced nodulation-suppressing CLE peptide encoding genes of *L. japonicus* and *M. truncatula*, known as LjCLE-RS1, LjCLE-RS2, LjCLE-RS3, MtCLE12 and MtCLE13^27–29,39,40^, cluster with these Group VI members of soybean and common bean^3^. Interestingly, two additional CLE prepropeptides of unknown function, called MtCLE35 and LjCLE5, also group closely (Supplementary Fig. S3). Okamoto *et al*.^27^ noted that LjCLE5 did not have a predicted signal peptide and that no expression could be detected. However, upstream of the previously predicted LjCLE5 start codon is another possible methionine (Fig. 5). The sequence following this alternative start codon corresponds closely with that of MtCLE12 (71.1% similarity), but the translation would result in a truncated protein prior to the CLE domain. Signal peptide prediction using SignalP (www.cbs.dtu.dk/services/SignalP/) suggests that there is a possible cleavage site at position 30 of the longer (but non-functional) LjCLE5. Interestingly, MtCLE35 contains the consensus sequence TLQAR, which is consistent with the nodulation-suppressing CLE peptides, whereas LjCLE5 does not. The functional analysis of MtCLE35 would be of great interest to the nodulation field.

In addition to having rhizobia-induced CLE peptides, soybean has an additional nitrate-induced CLE peptide, GmNIC1a, which acts locally to supress nodulation^39^. To date, no orthologue of GmNIC1a has been reported in *L. japonicus* or *M. truncatula*. Here, we used *GmNIC1a* and a BLAST search of the *L. japonicus* and *M. truncatula* genomes to reveal likely orthologous candidates (Supplementary Fig. S3). In soybean and common bean, *NIC1* and *RIC1* are located tandemly within the genome^39,40^. In *L. japonicus*, the putative NIC1 and RIC1 orthologues (*LjCLE40* and *LjCLE-RS2*, respectively) appear in tandem with LjCLE-RS3 and are approximately 24 kb apart on chromosome 3. Interestingly, LjCLE40 was also recently found to be induced by rhizobia inoculation^29^. In *M. truncatula*, the predicted orthologue of *NIC1* is *MtCLE34*, which is located tandemly on chromosome 2 with *MtCLE35*. However, a C > T mutation at base 148 of *MtCLE34* results in a premature stop codon and thus the translated product of this gene is likely non-functional. Further investigations are required to determine if the product is indeed truncated.

**Figure 7:**
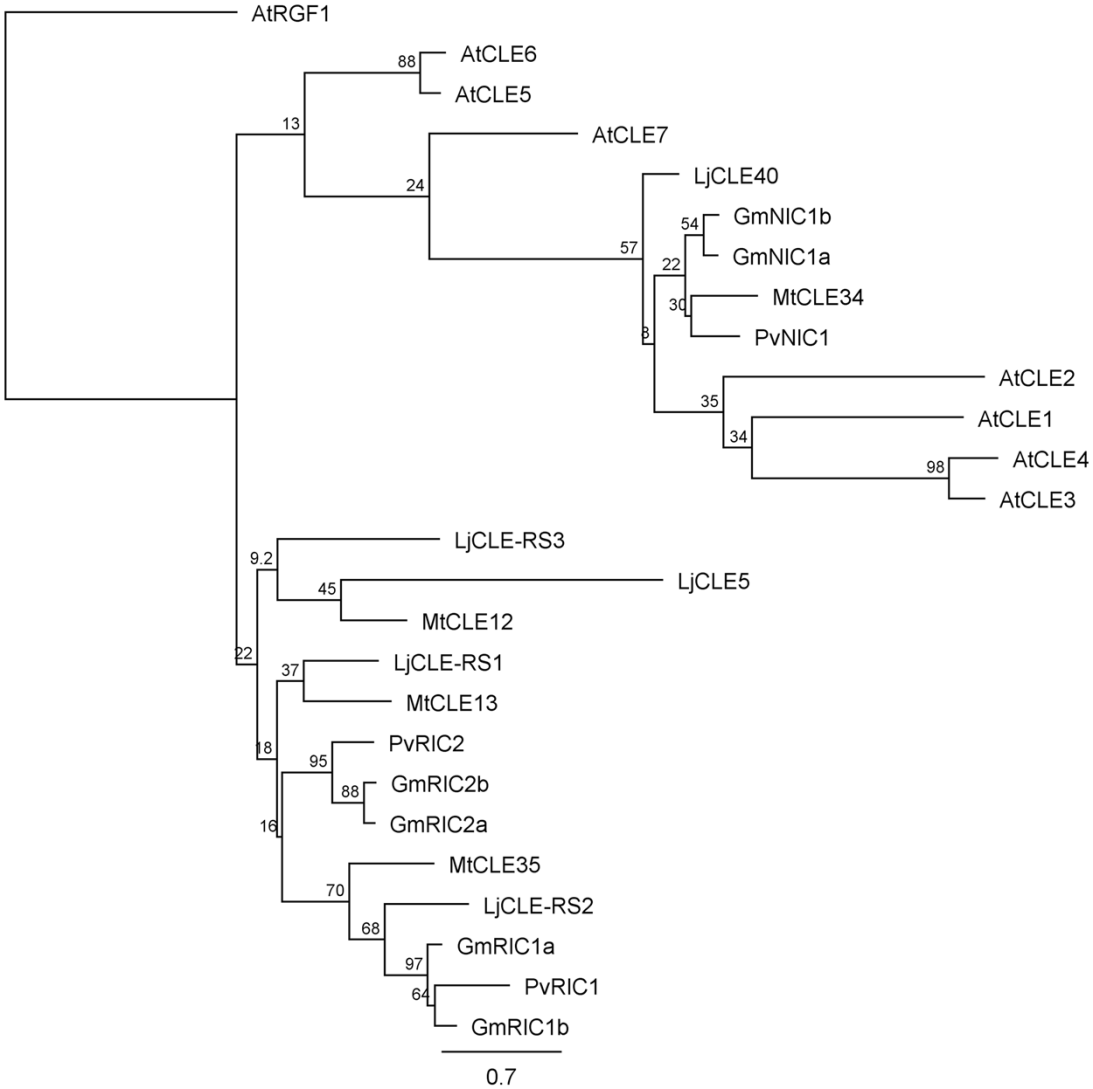
Phylogenetic tree of known legume nitrate-induced CLE peptides, rhizobia-induced CLE peptides, including two likely orthologous identified here in addition to *Arabidopsis thaliana* AtCLE1-7, which are most similar to these legume-specific CLE peptides. Bootstrap confidence values displayed are expressed as a percentage of 1,000 bootstrap replications, using AtRGF1 as an outgroup.

The legume nodulation CLE peptides are most similar to AtCLE1-7 of *A. thaliana*, however no direct orthologues have been identified as *A. thaliana* lacks the ability to form a symbiotic relationship with rhizobia or arbuscular mycorrhizae^2^. A targeted phylogenetic analysis was utilised here to investigate whether there are specific *A. thaliana* CLE peptides within AtCLE1-7 that are more closely linked with the nodulation CLE peptides of *M. truncatula*, *L. japonicus*, *P. vulgaris* and *G. max* (Fig. 7). As expected, the rhizobia-induced CLE peptides form a distinct branch from the nitrate-induced CLE peptides of legumes, and not surprisingly, the *A. thaliana* CLE peptides AtCLE1-7 group closer to these nitrate-induced sequences. This finding further supports the distinction of Group VI made by Hastwell *et al*. (2015).

### Expression of CLE peptide-encoding genes of *M*. *truncatula* and *L*. *japonicus*

It would be of little biological relevance to apply the peptides identified here to plants without first understanding their structural modifications and location of synthesis. We therefore used an *in-silico* approach to further assist in the functional characterisation of these genes. Publicly available transcriptome databases of *M. truncatula* and *L. japonicus* were used to collect expression data of the CLE peptide-encoding genes. A meta-analysis was performed to determine if putative orthologues identified by sequence characterisation and phylogenetic analyses exhibited similar expression patterns (Tables 2 and 3). Some similarity was seen between the putative orthologues, but the number of currently annotated CLE-peptide encoding genes limited a more detailed analysis.

**Table 2:**
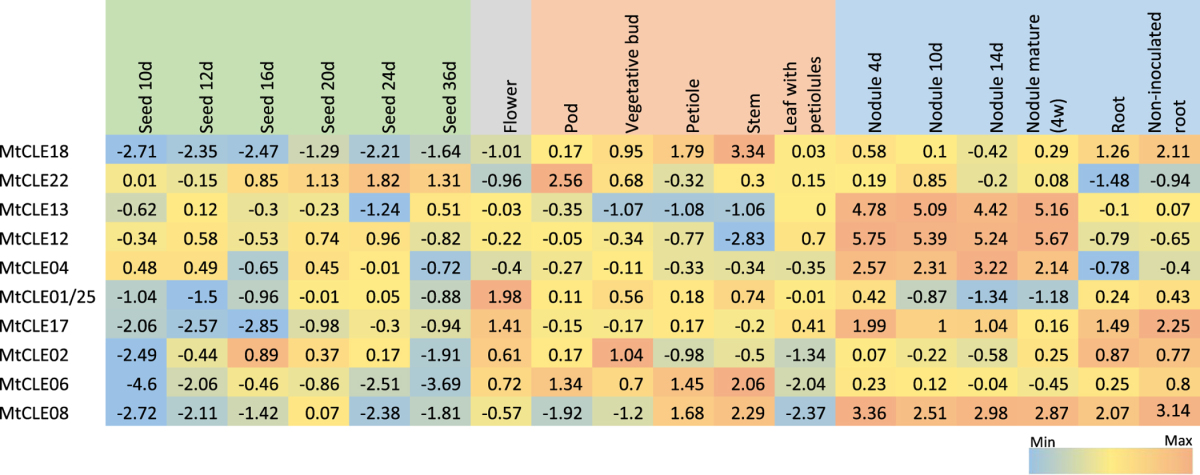
Normalised *Medicago truncatula* CLE peptide-encoding gene expression displayed as log2-transformed values (5.75 = 54.1 fold). The colour scale is independent for each gene.

**Table 3:**
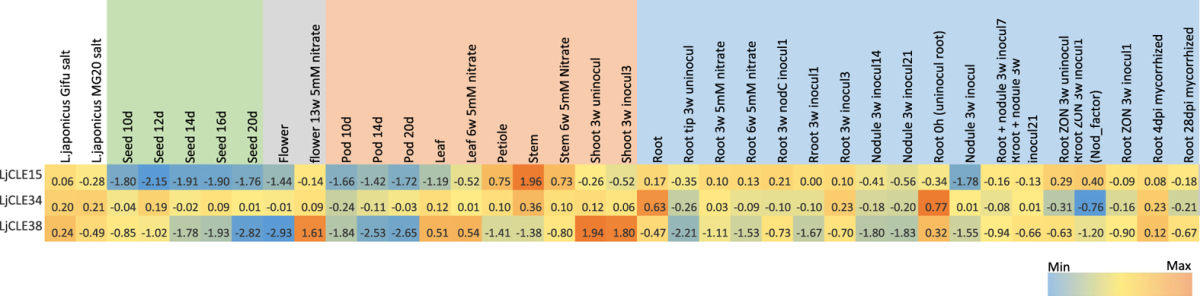
Normalised *Lotus japonicus* CLE peptide-encoding gene expression displayed as displayed as log2-transformed values (1.96 = 3.9 fold). The colour scale is independent for each gene.

A number of putative orthologues identified in the phylogenetic tree (Supplementary Fig. S3) showed similar expression trends across tissues, such as *PvCLE25* ^3^ and *MtCLE08*, which were both expressed in the root, nodules and stem (Table 2). LjCLE15 is expressed highest in the stem with lower expression levels found across all other tissue types and genes that group closely, *MtCLE18* and *PvCLE24*, are expressed in both the stem and root, whereas *AtCLE12*, which also groups closely is only found in the root (Tables 2 and 3). *MtCLE17* shares a similar expression pattern to *PvCLE23*, *GmCLE23a* and *GmCLE23b* ^3^, being expressed across all tissue types except in seeds, with *MtCLE17* also having notable higher expression in flowers than that of its putative orthologues, which shows little expression in the flower tissue (Table 2). *MtCLE12* and *MtCLE13* are currently the only functionally characterised *M. truncatula* CLE peptide-encoding genes, and the transcriptomic data for both genes is consistent with the literature^28^, being expressed in the nodules at different stages of development.

In contrast, some CLE peptide-encoding gene orthologues did not exhibit similar expression patterns within the transcriptomes according to the tissues and treatments available. *PvTDIF1*, *GmTDIF1a* and *GmTDIF1b* show high levels of expression across the different tissues^3^, with high root expression being of particular importance, as it is the only TDIF peptide-encoding gene to exhibit expression in the root. Their putative orthologues, *AtCLE41* and *AtCLE44* are also expressed in the root, in addition to other tissue types tested^3^, and *M. truncatula* orthologue, *MtCLE06*, shows no expression in the seeds and is only lowly expressed in the root. *PvCLE29* was noted by Hastwell *et al*.^3^ to have very high expression only in the flower. The putative orthologue *LjCLE19*, has previously been shown to respond in the root to phosphate treatment^30^ and more recently mycorrhizae colonization^31^, which is also not consistent with the expression of *PvCLE29* ^3^.

## Discussion

The importance of peptides in plant development is becoming increasingly evident with an extensive number of peptides and peptide families being discovered^1^. CLE peptides are no exception, with confirmed roles in meristematic tissue maintenance, and abiotic and biotic responses; however, the precise function of most is yet to be elucidated. To assist in the discovery of novel CLE peptide functions, the entire CLE peptide family of two model legumes, *M. truncatula* and *L. japonicus*, was identified here. Our analyses increased the number of annotated CLE peptides from 24 to 52 in *M. truncatula* and from 44 to 53 in *L. japonicus*. These were subjected to a range of comparative bioinformatics analyses to create a resource that can be utilised for further reverse-genetics-based functional characterisation. Additionally, six multi CLE domain-encoding genes and a number of pseudogenes were identified across the two species.

The phylogenetic analysis conducted using entire families of CLE prepropeptides of *M. truncatula*, *L. japonicus*, *A. thaliana* and *P. vulgaris* shows strong groupings between those having a similar CLE domain and a known or predicted function. The gene clusters identified here are generally conserved with those identified by Hastwell *et al*.^3^, which were divided into seven groups (Group I – VII).

*M. truncatula* and *L. japonicus* have a similar sized genome (500 Mbp) and share a common ancestor ~37-38 MYA, which is more recent than their shared ancestry with *P. vulgaris* (~45-59 MYA)^41^. The number of CLE peptide-encoding genes present (52 and 53 respectively), is consistent with the number in the *P. vulgaris* genome, 46, and is roughly half that of *G. max*, which has 84^3^ due to a more recent (~13 MYA) whole genome duplication event^42^.

The number of CLE peptide-encoding genes in the legumes is higher than that of *A. thaliana*, which has 32. This is predominately due to the absence of CLE peptide-encoding genes involved in symbioses between rhizobia (Group VI) or mycorrhizae^3,31,43^. The symbioses formed by legumes enable them to acquire nutrients that would otherwise be unavailable^44,45^. Nodulation control pathways are well characterised in *M. truncatula* and *L. japonicus*, beginning with the production of a CLE peptide^2,19,46^. However, a separate nitrate-regulated nodulation pathway identified in *G. max* has not yet been established in these two species. Here, a putative orthologue of *GmNIC1* and *PvNIC1*, which responds to the level of nitrate in the rhizosphere to inhibit nodulation^2,39,40^, has been identified in *M. truncatula*. However, *MtCLE34* is likely to be non-functional as a result of a truncation before the CLE domain. The putative orthologue in *L. japonicus*, *LjCLE5*, which has not yet been detected in gene expression studies, is likely to be non-functional as a result of a naturally-occurring insertion/deletion mutation. Further analysis is also needed to determine if MtCLE35 has a functional role in nodulation and if another gene in *L. japonicus* has gained the ability to regulate nodulation in response to nitrogen. Indeed, the latter is hinted towards by the ability of *LjLCE-RS1* to be induced by both rhizobia and nitrate to control nodule numbers^2,27^.

Although *A. thaliana* does not enter into a symbiosis with either rhizobia or mycorrhizae, its genome contains orthologues to known symbiosis genes, such as *AtPOLLUX* ^47^. However, our work indicates that no CLE peptide-encoding genes have yet been identified that show homology or synteny to the rhizobia-induced CLE peptides. It would be of interest to determine if such CLE peptide encoding genes previously existed, or exist but have been overlooked in *A. thaliana* due to being highly divergent from the symbiosis CLE peptides in legumes and other species.

Recent advances in genome sequencing, bioinformatics resources and the identification of entire CLE peptide families of soybean, common bean and Arabidopsis, have been utilised to capture the entire CLE peptide-encoding gene families of two important model legume species, *M. truncatula* and *L. japonicus*. Further characterisation of these CLE peptide-encoding genes revealed orthologues amongst the species, many of which appear functional, with some likely to be pseudogenes. The identification and genetic characterisation of these genes will benefit future studies aimed at functionally characterising these integral molecular components of plant meristem formation and maintenance.

## Methods

### Gene Identification

Candidate CLE peptide-encoding genes were identified in *L. japonicus* and *M. truncatula* using TBLASTN searches with known all CLE prepropeptides of *G. max* ^3^, *P. vulgaris* ^3^ and *A. thaliana* ^48^. The *M. truncatula Mt4.0v1* genome was searched in Phytozome (https://phytozome.jgi.doe.gov/)^49,50^ and the *L. japonicus v3.0* genome was searched in Lotus Base (https://lotus.au.dk/). Initial searches were conducted with E-value = 10. The results were manually validated for the presence of a CLE peptide-encoding gene in an open reading frame. Orthologues were also identified using TBLASTN of newly identified CLE prepropeptide sequences where clear orthologous were not identified between *M. truncatula* and *L. japonicus*, using E-value = 1.

Hidden Markov Models (HMMs) were generated for *M. truncatula* and *L. japonicus* CLEs individually, using all full length prepropeptide sequences as input into HMMER3, respectively (www.hmmer.org). Next, based on the generated HMMs, jackHMMER (www.hmmer.org) was applied to iteratively search for CLE sequences in *M. truncatula* and *L. japonicus* protein databases using a bit score of 50.

### Phylogenetic analysis

Multiple sequence alignments were constructed as outlined in Hastwell *et al*.^3^. Manual adjustments were made to some predicted sequences, particularly in regards to their start codon, based on similarity to duplicate genes, clustering genes, and/or likely orthologous genes. Multiple sequence alignments constructed without truncated or likely non-functional CLE prepropeptides were used to generate phylogenetic trees. The trees were constructed using methods described in Hastwell *et al*.^3^ using 1,000 bootstrap replications in all cases, except for the tree constructed using the entire families of *L. japonicus*, *M. truncatula*, *A. thaliana* and *P. vulgaris* CLE peptides, which used 100 bootstrap replications. Where orthologues were not apparent, the genomes of *L. japonicus* and *M. truncatula* were re-searched in an attempt to identify a possible orthologue.

### Sequence Characterisation

The presence of a signal peptide encoding domain and putative signal peptide cleavage site of the CLE prepropeptides was identified using SignalP (http://www.cbs.dtu.dk/services/SignalP /)^51^. If no signal peptide was detected, the sequence was manually examined for an up- or downstream methionine, which could be the likely start codon. The modified sequence was re-entered into SignalP and a signal peptide was detected in most instances. Possible intron boundary sites were identified using the NetPlantGene Server (http://www.cbs.dtu.dk/services/NetPGene/)^52,53^ and the nucleotide splice sites and resulting prepropeptides were compared with orthologous sequences. Sequence logo graphs of the CLE domain were generated using multiple sequence alignments in Geneious Pro v10.0.2^53^.

Genomic environments were established using five up- and down-stream annotated genes in Phytozome and Lotus Base (https://phytozome.jgi.doe.gov/; https://lotus.au.dk/)^49,50^. Orthologues of individual genes within the genomic environment lacking functional family annotations were identified using BLAST within and between the two databases.

### *M*. *truncatula* and *L*. *japonicus* transcriptome meta-analysis

The meta-analysis of the normalised transcriptome data was done using publicly available data sets located on the Medicago eFP browser (http://bar.utoronto.ca/efpmedicago/)^49,54,55^ and the *Medicago truncatula* Gene Expression Atlas (http://mtgea.noble.org/v3/)^54,56^ for *M. truncatula*, and The *Lotus japonicus* Gene Expression Atlas (http://ljgea.noble.org/v2/)^57^ for *L. japonicus*.

## Electronic supplementary material

Supplementary Information
