# Author Correction: CLE peptide-encoding gene families in *Medicago truncatula* and *Lotus japonicus*, compared with those of soybean, common bean and Arabidopsis

**DOI:** 10.1038/s41598-017-14991-9

**Published:** 2017-11-13

**Authors:** April H. Hastwell, Thomas C. de Bang, Peter M. Gresshoff, Brett J. Ferguson

**Affiliations:** 1 Centre for Integrative Legume Research, School of Agriculture and Food Sciences, The University of Queensland, St Lucia, Brisbane, Queensland 4072 Australia; 2 Plant Biology Division, Noble Research Institute LLC, Ardmore, Oklahoma 73401 USA; 3 Present Address: Department of Plant and Environmental Sciences, Section for Plant and Soil Sciences, Faculty of Science, University of Copenhagen, Thorvaldsensvej 40, DK-1871 Frederiksberg C, Denmark

*Scientific Reports* **7**:9384; doi:10.1038/s41598-017-09296-w; Article published online 24 August 2017

This Article contains an error in the legend of Figure 5.

“Multiple sequence alignment of the prepropeptides of AtCLE18 and LjCLE34. CLE domains are highlighted with a red box and the CLEL domain is underlined in blue. Conservation between amino acid residues of the two sequences is represented by grey (partial) and black (100%) shading”.

should read:

“Sequence alignment of *LjCLE5* and *MtCLE12*. The *LjCLE5* translation start site corresponding to that of *MtCLE12* results in a truncated prepropeptide (denoted by the asterisk shaded in black). The blue box represents an alternative start codon that results in the CLE domain region of LjCLE5 (underlined in red) being translated in frame. Black highlighted nucleic acid bases are 100% conserved”.

