# Supplementary material for: CLE peptide-encoding gene families in Medicago truncatula and Lotus japonicus, compared with those of soybean, common bean and Arabidopsis
Source: Sci Rep. 2017 Aug 24;7:9384. doi: 10.1038/s41598-017-09296-w (PMC5570945; doi:10.1038/s41598-017-09296-w)
Supplement: Supplementary file 1 — Supplementary Information [file 41598_2017_9296_MOESM1_ESM.pdf]

CLE peptide-encoding gene families in *Medicago truncatula* and *Lotus japonicus*, compared with those of soybean, common bean and Arabidopsis

Authors: April H. Hastwell[a], Thomas C. de Bang[bc], Peter M. Gresshoff[a], Brett J. Ferguson[a]*

Supplementary Figures and Tables

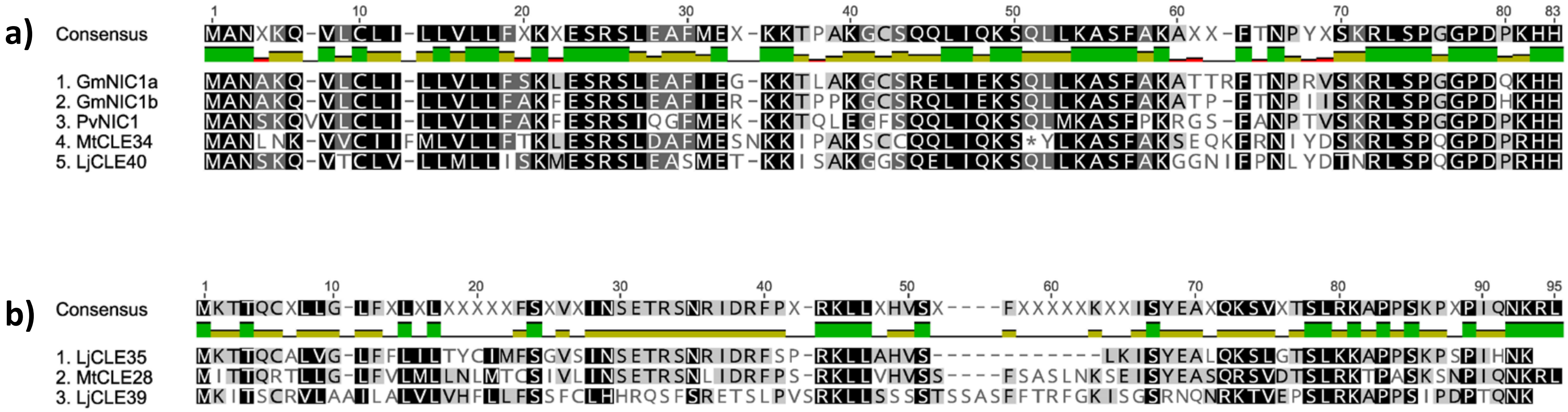

Supplementary Figure S1. Multiple sequence alignment of CLE prepropeptides. **a)** The likely pseudogene MtCLE34 and its orthologues in *Glycine max*, *Phaseolus vulgaris* and *Lotus japonicus*. **b)** LjCLE35, LjCLE39 and MtCLE28, which have non-canonical CLE domains. Grey nucleotides are semi-conserved and black nucleotides are 100% conserved.

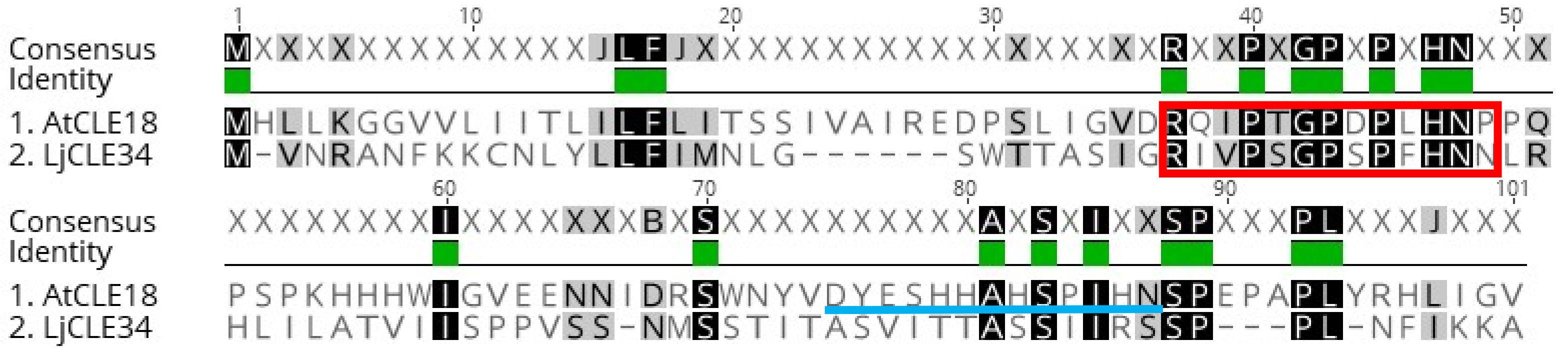

Supplementary Figure S2. Multiple sequence alignment of the prepropeptides of AtCLE18 and LjCLE34. CLE domains are highlighted with a red box and the CLEL domain is underlined in blue. Conservation between amino acid residues of the two sequences is represented by grey (partial) and black (100%) shading.

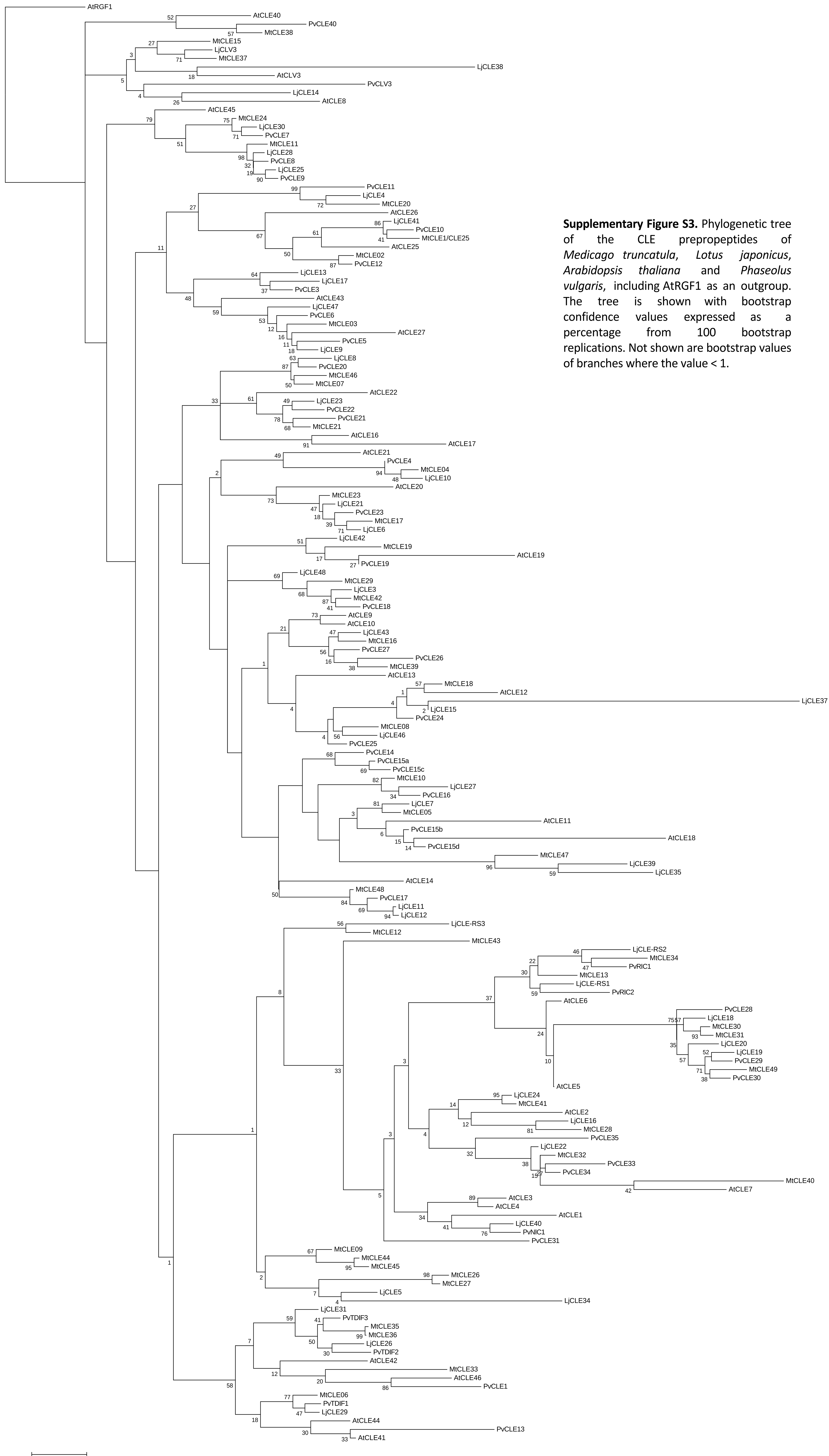

Supplementary Figure S3. Phylogenetic tree of the CLE prepropeptides of *Medicago truncatula*, *Lotus japonicus*, *Arabidopsis thaliana* and *Phaseolus vulgaris*, including AtRGF1 as an outgroup. The tree is shown with bootstrap confidence values expressed as a percentage from 100 bootstrap replications. Not shown are bootstrap values of branches where the value < 1.

a)

b)

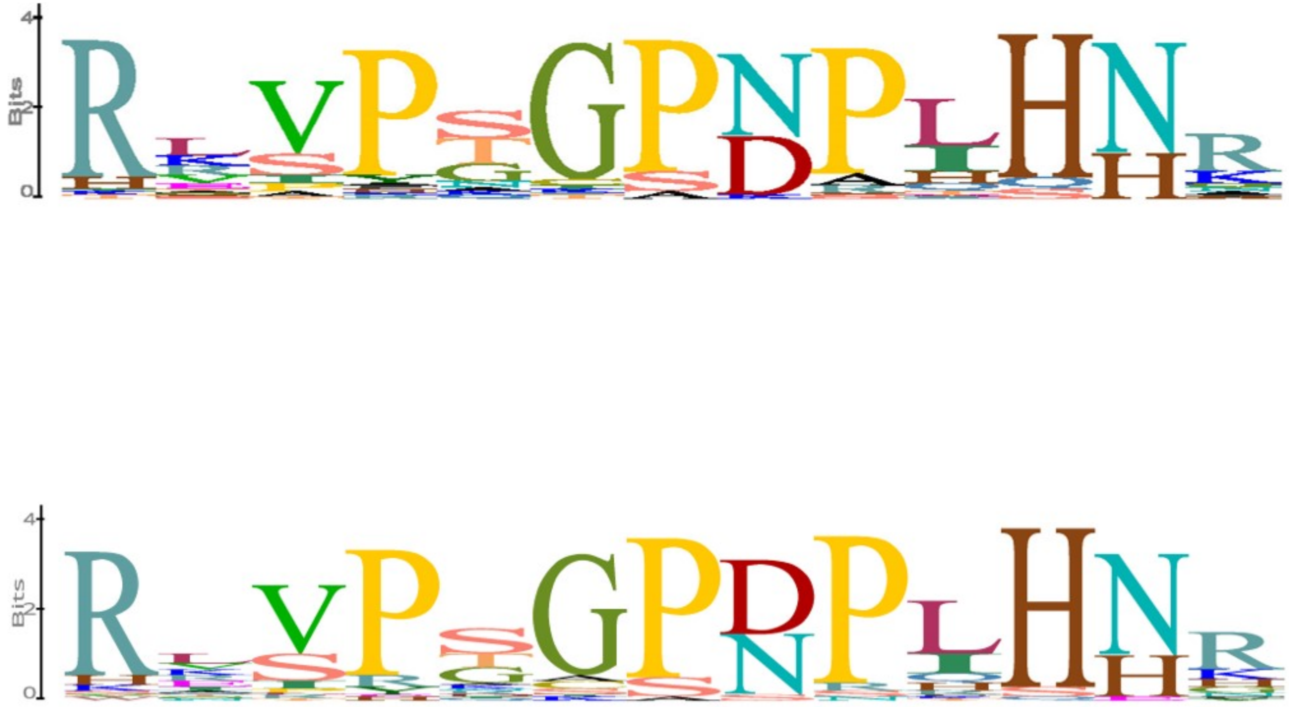

Supplementary Figure S4. Logo diagrams generated from the 12-13 amino acid CLE domains of multi-CLE domain encoding genes of **a)** *Medicago truncatula* and **b)** *Lotus japonicus*.

Supplementary Table S1. CLE domain sequences of *Medicago truncatula* and *Lotus japonicus* with references to their initial identification.

Name	CLE domain sequence	Reference	Name	CLE domain sequence	Reference
MtCLE1/CLE25	RVPNGPDPIHNR	Oelkers et al. 2008	LjCLE-RS1	RLSPGGPDPQHNG	Okamoto et al. 2009
MtCLE02	RRVPNGPDPIHNR	Oelkers et al. 2008	LjCLE-RS2	RLSPGGPDPQHNN	Okamoto et al. 2009
MtCLE03	RKVPSCPDPLHN	Oelkers et al. 2008	LjCLE-RS3	WISPGGPDPKHNK	Nishida et al. 2016
MtCLE04	RGVPSGANPLHNR	Oelkers et al. 2008	LjCLV3	RKVPSGPDPLHHH	Okamoto et al. 2009
MtCLE05	HVVPSGPNPLHN	Oelkers et al. 2008	LjCLE3	RVVPTGPNPLHNR	Okamoto et al. 2009
MtCLE06	HEVPSGPNPISNR	Oelkers et al. 2008	LjCLE4	RKVPDASDPLHNR	Okamoto et al. 2009
MtCLE07	RKVYTGPNPLHNR	Mortier et al. 2010	LjCLE5	RLSPGGPNHIHNN[a]	Okamoto et al. 2009
MtCLE08	RLVPTGPNPLHH	Oelkers et al. 2008	LjCLE6	RRVPTGSNPLHNK	Okamoto et al. 2009
MtCLE09	RLVPTGPNRMHNR	Mortier et al. 2010	LjCLE7	RVVPSGPNRLHN	Okamoto et al. 2009
MtCLE10	TSTPGGPNPLHN	Mortier et al. 2010	LjCLE8	RKVYTGPNPLHNR	Okamoto et al. 2009
MtCLE11	RTVKKGSNPIHNR	Oelkers et al. 2008	LjCLE9	RKVPSCPDPLHN	Okamoto et al. 2009
MtCLE12	RLSPGGPNHIHN	Oelkers et al. 2008	LjCLE10	RGVPSGANPLHNR	Okamoto et al. 2009
MtCLE13	RLSPAGPDPQHNG	Cock and McCormick 2001	LjCLE11	RQVPGGPNPLHHG	Okamoto et al. 2009
MtCLE14	[b]	Mortier et al. 2010	LjCLE12	RQVPSGPNPLHNR	Okamoto et al. 2009
MtCLE15	RKVPTGPDPLHHH	Mortier et al. 2010	LjCLE13	REVPSSPDPLHNR	Okamoto et al. 2009
MtCLE16	RRVPTGPNPLHN	Cock and McCormick 2001	LjCLE14	RKVPTGPNPLHEH	Okamoto et al. 2009
MtCLE17	RKVPTGSNPLHNK	Oelkers et al. 2008	LjCLE15	RRVPTGPNPLHH	Okamoto et al. 2009
MtCLE18	RRVPTGPNPLHH	Cock and McCormick 2001	LjCLE16	RLSPGGPDPRHH	Okamoto et al. 2009
MtCLE19	RQVPSSPDPLHNR[a]	Mortier et al. 2010	LjCLE17	RAVPSSPDRLHNR	Okamoto et al. 2009
MtCLE20	RRVPNTSDPLHNR	Cock and McCormick 2001	LjCLE18	RVSPAGPDPQHH	Okamoto et al. 2009
MtCLE21	RKIYTGPNPLHNR	Cock and McCormick 2001	LjCLE19	RVSPAGPDPQHH	Okamoto et al. 2009
MtCLE22	[b]	Oelkers et al. 2008	LjCLE20	RVSPEGPDSQHH	Okamoto et al. 2009
MtCLE23	RRVPTGANPLHNK	Mortier et al. 2010	LjCLE21	RMVPTGSNPLHNK	Okamoto et al. 2009
MtCLE24	RRVRKGSDPIHNR	Mortier et al. 2010	LjCLE22	RLSPGGPDPHHH	Okamoto et al. 2009
MtCLE26	[b]		LjCLE23	RKIYTGPNPLHNR	Okamoto et al. 2009
MtCLE27	[b]	[c]	LjCLE24	RVSPGGPDSHHHF	Okamoto et al. 2009
MtCLE28	KTPASKSNPIQNK		LjCLE25	RTVRKGSDPIHNR	Okamoto et al. 2009
MtCLE29	RLSPGGPDPRHH	[c]	LjCLE26	HEVPSGPNPISNR	Okamoto et al. 2009
MtCLE30	RHVPTGPNPLHNR	[c]	LjCLE27	SNTPGGPNPLHN	Okamoto et al. 2009
MtCLE31	RVAPAGPDSQHH	[c]	LjCLE28	RTVRKGSDPIHNR	Okamoto et al. 2009
MtCLE32	RVAPSGPDAHHH	[c]	LjCLE29	HEVPSGPNPISNR	Okamoto et al. 2009
MtCLE33	RLSPSGPDPHHH	[c]	LjCLE30	RRVRRGSDPIHNR	Okamoto et al. 2009
MtCLE34	RLSPQGPDPRHH[a]	[c]	LjCLE31	HEVPSGPNPISNR	Okamoto et al. 2009
MtCLE35	RLSPGGPDPHHHA	[c]	LjCLE32	[b]	Okamoto et al. 2009
MtCLE36	REVPSGPNPISNR	[c]	LjCLE33	[b]	Okamoto et al. 2009
MtCLE37	HEVPSGPNPISNR	[c]	LjCLE34	RIVPSGPSPFHNN	Okamoto et al. 2009
MtCLE38	RKIPSGPDPLHHN	[c]	LjCLE35	KAPPSKPSPIHNK	Okamoto et al. 2009
MtCLE39	REVPTGPDPLHHN	Hastwell et al. 2015	LjCLE37	REREIAPDPLHHY	Okamoto et al. 2009
MtCLE40	RLVPSGPNPLHN	[c]	LjCLE38	SQPPNTARAAHDD	Okamoto et al. 2009
MtCLE41	RLIHTGPNPLHN	[c]	LjCLE39	KAPPSIPDPTQNK	Nishida et al. 2016
MtCLE42	RLSPGGPDAHHH	[c]	LjCLE40	RLSPQGPDPRHH	Nishida et al. 2016
MtCLE43	RVSPGGPDAHHHF		LjCLE41	RRVPNGPDPIHNR	Nishida et al. 2016
MtCLE44	RVVPTGPNPLHNR	[c]	LjCLE42	RIIHTGPNPLHN	Nishida et al. 2016
MtCLE45	RKVPSCPDPLHN	[c]	LjCLE43	RRVPTGPNPLHN	
MtCLE46	RLSPGGPDRHHN		LjCLE44	RRVPNGPDPIHNR	
MtCLE47	RLVPQGPNPIHNR		LjCLE45	RIVPSGPNPLHN	
MtCLE48	RLVPQGSKPIHNG		LjCLE46	[b]	
MtCLE49	IAPPSIPNPTQNK		LjCLE47	[b]	
MtCLE50	RKVFTGPNPLHNR	[c]	LjCLE48	[b]	
MtCLE51	RIPPSRPNPTQNK		LjCLE49	RLSPGGPDPHHH	
MtCLE52	RDVPGGPNPLHN	[c]	LjCLE50	RLVPSGPNPLHH	
MtCLE53	RVSPGGPDAQHH	[c]	LjCLE51	RVIPSCPDPLHN	
			LjCLE52	RLVPTGPNPLHNR	

[a]CLE domain unlikely to be translated, see Supplementary Fig. S1

[b]Multi-CLE domain prepropeptide, see Fig. 3 and Supplementary Table. S3

[c]Follwing our identification of this gene, it was independently found via a multi-species bioinformaticanalysis of CLE peptides (Goad et al. 2016), but was not names and no sequence analyses were performed.

Supplementary Table S2. Amino acid sequence similarity (%) amongst tandem repeat CLE prepropeptides of **a)** *Medicago truncatula* and **b)** *Lotus japonicus*. Grey boxes highlight similarities between genes located in tandem with each other.

a)

	MtCLE12	MtCLE13	MtCLE14	MtCLE26	MtCLE27	MtCLE31	MtCLE32	MtCLE33	MtCLE34*	MtCLE35	MtCLE36	MtCLE37	MtCLE46	MtCLE47	MtCLE48
MtCLE09	16.1	15.2	20.1	10.2	13.9	18.5	15.4	18.9	23.4	17.4	15.6	11.9	10.9	57.1	52.8
MtCLE12	-	30.6	17.6	15.1	11.5	21.6	18.4	22.4	21.2	24.4	18.5	17.6	21.4	25.6	18.2
MtCLE13	-	-	15.4	13.0	11.4	29.1	22.4	19.5	24.6	31.2	8.4	9.3	13.7	19.3	12.6
MtCLE14	-	-	-	17.3	21.8	16.6	16.3	20.0	16.1	20.3	18.5	17.8	21.2	19.4	29.1
MtCLE26	-	-	-	-	79.5	12.3	15.7	14.2	10.5	13.2	10.5	9.6	13.8	15.1	16.1
MtCLE27	-	-	-	-	-	9.3	15.9	13.5	9.6	8.8	16.4	13.3	11.4	17.2	12.4
MtCLE31	-	-	-	-	-	-	77.6	29.9	29.1	23.9	18.5	17.8	18.6	17.6	18.2
MtCLE32	-	-	-	-	-	-	-	29.4	17.0	23.3	14.2	13.2	19.4	17.8	11.6
MtCLE33	-	-	-	-	-	-	-	-	14.5	25.9	14.0	13.9	22.1	20.8	21.7
MtCLE34	-	-	-	-	-	-	-	-	-	20.4	18.1	18.1	18.5	17.0	11.0
MtCLE35	-	-	-	-	-	-	-	-	-	-	8.3	15.7	28.0	19.7	13.5
MtCLE36	-	-	-	-	-	-	-	-	-	-	-	95.4	18.7	16.1	13.0
MtCLE37	-	-	-	-	-	-	-	-	-	-	-	-	20.6	14.5	13.0
MtCLE46	-	-	-	-	-	-	-	-	-	-	-	-	-	37.7	22.2
MtCLE47	-	-	-	-	-	-	-	-	-	-	-	-	-	-	81.6

*MtCLE34 is truncated

b)

	LjCLE12	LjCLE19	LjCLE20	LjCLE33	LjCLE40	LjCLE46	LjCLE47	LjCLE-RS2	LjCLE-RS3
LjCLE11	91.0	18.7	18.5	27.8	26.4	13.3	18.1	20.2	17.0
LjCLE12	-	15.4	17.9	20.0	20.0	18.8	17.6	14.1	18.3
LjCLE19	-	-	57.0	12.9	30.7	12.1	15.2	19.8	20.2
LjCLE20	-	-	-	10.5	33.7	12.9	13.8	19.8	22.9
LjCLE33	-	-	-	-	8.9	58.4	53.1	13.5	13.6
LjCLE40	-	-	-	-	-	10.5	11.2	24.7	24.7
LjCLE44	-	-	-	-	-	-	86.3	12.7	13.7
LjCLE45	-	-	-	-	-	-	-	9.3	16.3
LjCLE-RS2	-	-	-	-	-	-	-	-	22.8

Supplementary Table S3. Amino acid residue conservation around the CLE domains (in bold) of multi-CLE domain prepropeptides.

	LjCLE32	LjCLE33[a]	LjCLE46	LjCLE47	MtCLE14[b]	MtCLE22	MtCLE26	MtCLE27
	IDRIT**RESPGGPDPRHHR**SFQPSN	SNGTN**RLVSTGPNRAESP**STGPVAAHSFVSKDFGF	AKYLVPNDIGFNYEIN**RLVPSGPNQEQSP**DPVPVV	VLSLAVNDRVTANGIN**RLVLSGPNR**	FPDNVVKNDLKGVYPNHNGFLQFE	EIGEL**RKVPSSPDPIHNS**DIDSIEDENKS	VVSDRLTSYNIK**RKVPTGPNSQLSP**DPVPIMAM	VADRVTSYNIK**RKVPTGPNPQLSP**DPVPVMAM
	VNHIT**RESPGGPDPRHHH**SLRPSN	NHEIE**RLVPTSPNPAQSS**GSGTIVV	AHSFGSKDFGFNHEIK**RLVPSGPNQAQSP**DPVPVV	AKYLVPNDIGFNYEIN**RLVPSGPNQEQSP**DPVPVV	NVYRTT**RLSPGVPDPYHLR**SLQSE	QIGGL**RKVPPYAHPIHKN**VADSYRVEKKS	STYYNFIREIK**RKVPTGPNPQLSP**DPVPVMAM	TTNYNFIHEIK**RKVPTGLNPTQSP**DPVPAVER
	VYHIT**RESPGGPDPRHHH**SLQPNN	NNGIQ**RLITTGPNAAQSP**DPGPVVAHSFVPKDFGF	AHSFVSKDFGFNHGIE**RLVPSSPNHTQSP**DSIPVL	AHSFGSKDFGFNHEIK**RLVPLGPNQAQSP**DPVPIV	NVHRIA**RLSPGGPDPHHHN**SLRPE	QIRGL**TKVPTSPDPIHNS**DSVSVEDENKP	SNDYKNFNHVIK**RKVPTGPNSAQSP**DPVPNVKP	SNDCNFNHVIK**RKVLTGPNPAQSP**DLVPNVKP
	VYHIT**RESPEGPDPRHHH**SFQPSN	NLEIK**RLVPTSPNSAQSQ**DSPVVAHSFVSKDFGF	TRSFSSMDFDFNHEIK**RLVPSGPNQEQSP**DPVPVV	AHSFVSKNFDFNHEIK**RLVPSGPNQEQSP**DPVPVV	NVHRIA**RLSPGGPDPHHHN**FLLSE	QIGRA**RMVSSGPNPLHNR**LINSVGTKNMP		
	VYHIT**RESPGGPDPRHHH**SLRPSN	NHKIN**RLVPTGPNPTKSP**DPPIVVHSFGSKDFGF	AHSFVSKDFGFNHEIE**RLVPSGPSHAQSP**DPVPVV	AHSFVSKDFGFNHEIE**RLVPSGPSHAQSP**DPVPVV	NVHHIA**RLSPGGPDPHHHN**SLRPE			
	VYHIT**RESPGGPDPRHHH**SFQPGN	NHEIK**RLVPTGPNPAHSP**DPRPVVAYTFASNNLGL	ARSFVFKDFGFNYEIK**RLIPSGPNQAQSP**DPVPVV	ARSFVFKDFGYNYEIK**RLIPSGPNQAQSP**DPVPVV	NVHRIA**RLSPGGPDPHHHN**CLQLE			
	VYHIT**RESPEGPDPRHHH**SLQPNN	NHEIK**RLVPTCQDPTQSP**DPTHVMAHSFVSKDFGL	ARSFVSKGSDFNHEIK**RHIPSDLNQAQSR**YPIPVV	ARSFVSKGSDFNHEIK**RHIPSDLNQAQSR**YPIPVV	NVHRIS**RPSPGGLDPCHNF**LQHG			
	VYHIT**RESPGGPDPRHHH**SLQPNY	NHEIK**RLVPTCPNTTQSP**DPGPDVAHSFVTKYFGF			NAYHIA**RFSPRGPNPYHHR**FLQP			
		NHEIK**RIVPTGSNPAPSP**DPGPNVTHSCVTKCFGF						
		NRKIKRFIPFMSKSSTIT						
Sequence conservation	: :****** *******:*::*.	. :*:: * : : * . .	:: : . .**: *:* :**. .: ** :**:	. : :. *:* : . .:	*.:: :* ** :* * *	:* * .*:*: * ::	********** ****** :	: : ***** ** ** *** ** :
Repeat length	24	25-35	35	25-35	23-24	29	32-33	32
No. of CLE domain number	8	9	7	7	7	4	3	3

[a]10th repeat is not part of the consensus sequence

[b]1st repeat is not part of the consensus sequence

Supplementary Table S4. Frequency (%) of amino acid residues in the CLE prepropeptides of *Lotus japonicus* and *Medicago truncatula*.

**Amino acid**	***L. japonicus***	***M. truncatula***
Ala	4.4	4.1
Cys	1.4	1.6
Asp	3.0	3.0
Glu	3.5	3.3
Phe	5.4	6.0
Gly	5.4	4.8
His	4.5	5.1
Ile	4.9	4.8
Lys	6.1	6.6
Leu	10.8	10.5
met	3.2	3.4
Asn	5.4	6.6
Pro	6.9	5.9
Gln	3.4	3.5
Arg	7.1	6.5
Ser	11.4	10.8
Thr	4.9	5.8
val	6.1	5.7
Trp	0.6	0.5
Tyr	1.5	1.5

Supplementary Table S5. CLE prepropeptides of *Medicago truncatula* and *Lotus japonicus* and how they cluster with soybean CLE prepropeptide Groups I - VII from Hastwell et al. (2015).

**Group I**		**Group II**		**Group III**		**Group IV**		**Group V**		**Group VI**		**Group VII**		**Undetermined**[a]	
LjCLV3	MtCLE15	LjCLE9	MtCLE1/CLE25	LjCLE26	MtCLE06	LjCLE7	MtCLE05	LjCLE3	MtCLE07	LjCLE-RS1	MtCLE12	LjCLE16	MtCLE14	LjCLE4	MtCLE09
LjCLE14	MtCLE22	LjCLE10	MtCLE02	LjCLE29	MtCLE36	LjCLE11	MtCLE10	LjCLE6	MtCLE08	LjCLE-RS2	MtCLE13	LjCLE18	MtCLE29	LjCLE33	MtCLE26
LjCLE48	MtCLE38	LjCLE13	MtCLE03	LjCLE31	MtCLE37	LjCLE12	MtCLE52	LjCLE8	MtCLE16	LjCLE-RS3	MtCLE35	LjCLE19	MtCLE31	LjCLE34	MtCLE27
	MtCLE39	LjCLE17	MtCLE04			LjCLE27		LjCLE15	MtCLE17	LjCLE5		LjCLE20	MtCLE32	LjCLE35	MtCLE28
		LjCLE24	MtCLE11					LjCLE21	MtCLE18			LjCLE22	MtCLE33	LjCLE37	MtCLE47
		LjCLE25	MtCLE19					LjCLE23	MtCLE21			LjCLE24	MtCLE34	LjCLE38	MtCLE48
		LjCLE28	MtCLE20					LjCLE42	MtCLE23			LjCLE32	MtCLE42	LjCLE39	MtCLE49
		LjCLE30	MtCLE24					LjCLE43	MtCLE30			LjCLE40	MtCLE43	LjCLE44	MtCLE51
		LjCLE41	MtCLE45					LjCLE50	MtCLE40			LjCLE49	MtCLE48	LjCLE45	
		LjCLE44							MtCLE41				MtCLE53	LjCLE46	
		LjCLE51							MtCLE44					LjCLE47	
									MtCLE50						

[a]CLE prepropeptides in this category did not clearly fit into any Group are predominately either multi-CLE domain prepropeptides or have non-canonical CLE domains.
